# Deguelin suppresses non-small cell lung cancer by inhibiting EGFR signaling and promoting GSK3β/FBW7-mediated Mcl-1 destabilization

**DOI:** 10.1038/s41419-020-2344-0

**Published:** 2020-02-21

**Authors:** Feng Gao, Xinfang Yu, Ming Li, Li Zhou, Wenbin Liu, Wei Li, Haidan Liu

**Affiliations:** 1 Department of Cardiovascular Surgery, The Second Xiangya Hospital of Central South University, 410011 Changsha, Hunan P.R. China; 2 Clinical Center for Gene Diagnosis and Therapy, The Second Xiangya Hospital of Central South University, 410011 Changsha, Hunan P.R. China; 3 Department of Ultrasonography, The Third Xiangya Hospital of Central South University, 410013 Changsha, Hunan P.R. China; 4 Department of Cancer Biology, Lerner Research Institute, Cleveland Clinic, 9500 Euclid Avenue, Cleveland, OH 44195 USA; 5 Changsha Stomatological Hospital, 410004 Changsha, Hunan P.R. China; 6 School of Stomatology, Hunan University of Chinese Medicine, 410208 Changsha, Hunan P.R. China; 7 Department of Pathology, Xiangya Hospital of Central South University, Changsha, 410008 Hunan P.R. China; 8 Department of Pathology, Hunan Cancer Hospital, 410013 Changsha, Hunan P.R. China; 9 Department of Radiology, The Third Xiangya Hospital of Central South University, 410013 Changsha, Hunan P.R. China

**Keywords:** Chemotherapy, Ubiquitylation

## Abstract

Activating mutations of epidermal growth factor receptor (EGFR) play crucial roles in the oncogenesis of human non-small cell lung cancer (NSCLC). By screening 79 commercially available natural products, we found that the natural compound deguelin exhibited a profound anti-tumor effect on NSCLC via directly down-regulating of EGFR-signaling pathway. Deguelin potently inhibited in vitro EGFR kinase activity of wild type (WT), exon 19 deletion, and L858R/T790M-mutated EGFR. The in silico docking study indicated that deguelin was docked into the ATP-binding pocket of EGFRs. By suppression of EGFR signaling, deguelin inhibited anchorage-dependent, and independent growth of NSCLC cell lines, and significantly delayed tumorigenesis in vivo. Further study showed that deguelin inhibited EGFR and downstream kinase Akt, which resulted in the activation of GSK3β and eventually enhanced Mcl-1 phosphorylation at S159. Moreover, deguelin promoted the interaction between Mcl-1 and E3 ligase SCF^FBW7^, which enhanced FBW7-mediated Mcl-1 ubiquitination and degradation. Additionally, phosphorylation of Mcl-1 by GSK3β is a prerequisite for FBW7-mediated Mcl-1 destruction. Depletion or pharmacological inactivation of GSK3β compromised deguelin-induced Mcl-1 ubiquitination and reduction. Taken together, our data indicate that enhancement of ubiquitination-dependent Mcl-1 turnover might be a promising approach for cancer treatment.

## Introduction

Activating mutations of epidermal growth factor receptor (EGFR), including a deletion in exon 19 and an L858R mutation in exon 21, are a driving force for some non-small cell lung cancer (NSCLC)^1–3^. Therapeutic strategies using tyrosine kinase inhibitors (TKI), such as gefitinib, to suppress EGFR kinase activity, have become first-line therapies for these patients and shown a significant clinical response. Unfortunately, most patients who initially respond to TKIs eventually develop acquired resistance within 1–2 years of treatment^4,5^. Several mechanisms of acquired resistance have been identified, and over one-half of the cases of first-generation TKI resistance were related to the T790M secondary mutation in exon 20 of EGFR. Also, c-MET amplification, overexpression or hyperactivation of other tyrosine kinase receptors, such as ErbB2 and insulin-like growth factor 1 receptor (IGF1R), and PIK3CA and/or K-ras mutations have also been identified to associate with acquired resistance^6–9^. Although TKI resistance has increased clinically, the treatment strategies to successfully overcome NSCLC TKI resistance are still limited.

Hyperactivation of EGFR signaling leads to increased proliferation and survival in human cancer cells. Previous studies showed that upregulation of the Bcl-2 family member, myeloid cell leukemia sequence 1 (Mcl-1), is involved in EGFR signaling-mediated tumor cell survival. EGF promotes Mcl-1 expression by increase Mcl-1 transcription in an Elk-1 transcription factor-dependent manner^10^. EGFR mutant NSCLC cells upregulate Mcl-1 through mTORC1-mediated mRNA translation, which contributes to EGFR TKI resistance^11^. Moreover, inhibition of EGFR by shRNA or erlotinib disrupts Bim binding to Mcl-1 and restoring the sensitivity to ABT-737^12^. In addition, TKI increase Mcl-1 degradation and, in combination with Bcl-XL/Bcl-2 inhibitors, drive prostate cancer apoptosis^13^. Even EGFR activation regulated Mcl-1 transcription was well studied, the mechanisms regarding EGFR signaling and Mcl-1 stability or ubiquitination need to be further elucidated.

Accumulating evidence reveals that the natural product deguelin exhibits profound anti-cancer potentials in multiple human cancer models, including lung cancer^14^, hepatocellular carcinoma^15^, colorectal cancer^16^, esophageal carcinoma^17^, metastatic melanoma^18^, and breast cancer^19^. The molecular mechanism studies showed that delay of cell cycle progression by inhibition of Aurora B kinase, regulation of metabolisms, such as suppression of glycolysis, down-regulation of angiogenesis and metastasis, and activation of intrinsic apoptosis were related to deguelin-mediated anti-tumor effect^20–22^. However, the effect of deguelin on EGFR signaling, as well as its inhibitory efficacy on both gefitinib sensitive and resistant cells, was not clear.

In the present study, we found that deguelin inhibited both EGFR WT and activating mutations cells in vitro and in vivo. Deguelin attenuated EGFR-Akt signaling and decreased the Mcl-1 protein level in a ubiquitination-dependent manner, which eventually resulted in the activation of apoptosis. Our data revealed that suppression of EGFR signaling and reduction of Mcl-1 might help to overcome resistance to targeted therapy.

## Materials and methods

### Reagents and antibodies

Compounds, including deguelin, gefitinib, MG132, PD98059, LY294002, and SB216763, were purchased from Selleck Chemicals (Houston, TX). The chemicals, including Tris, NaCl, and SDS for molecular biology and buffer preparation were purchased from Sigma (St. Louis, MO). Fetal bovine serum (FBS), cell culture medium, and supplements were from Invitrogen (Grand Island, NY). Primary antibodies against p-EGFR-Tyr1068 (#3777, 1:2000), EGFR (#4267, 1:2000), p-ERK1/2-Thr202/Tyr204 (#4370, 1:1000), ERK1/2 (#9102, 1:2000), p-Akt-Ser473 (#4060, 1:1000), Akt (#4691, 1:1000), PARP (#9532, 1:1000) cleaved-caspase 3 (#9664, 1:1000), Bcl-2 (#4223, 1:1000), Bcl-XL (#2764, 1:1000), Mcl-1 (#39224, 1:1000), GSK3β (#12456, 1:1000), p-GSK3β-Ser9 (#5558, 1:1000), ubiquitin (#3936, 1:1000), ubiquitin (#43124, 1:1000), α-Tubulin (#2125, 1:5000), β-actin (#3700, 1:5000), HA-tag (#2999, 1:3000), and His-tag (#12698, 1:3000) were purchased from Cell Signaling Technology, Inc. (Danvers, MA). Flag-tag (F3165, 1:5000) antibody was obtained from Sigma Aldrich (St. Louis, MO). Antibodies against p-Mcl-1-Ser159 (ab111574, 1:1000), FBW7 (ab109617, 1:1000), FBW7 (ab187815, 1:2000) were from Abcam (Cambridge, UK). Secondary antibodies, including anti-mouse IgG, HRP-linked antibody (#7074, 1:10,000) and anti-mouse IgG, HRP-linked antibody (#7076, 1:10,000), were obtained from Cell Signaling Technology, Inc. (Danvers, MA). Antibodies for immunohistochemistry staining (IHC), including anti-ki67 (ab15580, 1:300) and anti-p-Akt (ab81283, 1:100) were obtained from Abcam. Anti-p-EGFR (#3777, 1:100), anti-p-ERK1/2 (#4370, 1:100), and anti-Mcl-1 (#39224, 1:100) were purchased from Cell Signaling Technology, Inc.

### Cell culture and transfection

Human NSCLC cells, including A549 (EGFR WT), H3255 (EGFR L858R), H1975 (EGFR L858R/T790M), and HCC827 (EGFR Del E746-A750) and normal immortalized lung epithelial cells HBE and NL20, were purchased from American Type Culture Collection (ATCC, Manassas, VA). The cells were cultured at 37 °C in a humidified incubator with 5% CO_2_ according to ATCC protocols. All the NSCLC cells were subjected to mycoplasma analysis and cytogenetically tested and authenticated before being frozen. 293T cell was purchased from ATCC and maintained in DMEM medium supplemented with 10% FBS and 1% antibiotics. Ba/F3 cell was purchased form Cell Engineering Division/RIKEN BioResource Center (Tsukuba, Ibaraki, Japan) and maintained in RPMI1640 + 10% FBS + 10% WEHI-3 cell conditioned medium according to instructions provided. For transfection experiments, the Lipofectamine^®^ 2000 (Thermo Fisher) transfection reagent was used following the manufacturer’s instructions.

### Immunoblotting

Immunoblotting (IB) was performed as described previously^23^. Briefly, whole-cell lysates were extracted with RIPA buffer (10 mM Tris–Cl (pH 8.0), 1 mM EDTA, 0.5 mM EGTA, 1% Triton X-100, 0.1% sodium deoxycholate, 0.1% SDS, 140 mM NaCl) supplied with protease and phosphatase inhibitors. The cell lysate was subjected to protein concentration, followed by SDS–PAGE gel electrophoresis and antibody hybridization. The target proteins were visualized by chemiluminescence (Amersham Biosciences, Piscataway, NJ).

### MTS assay

NSCLC cells were seeded (2.5 × 10^3^/well/100 μL) into 96-well plates and treated with various concentrations of deguelin or gefitinib as indicated, cell viability was assessed by MTS assay (Promega, Madison, WI) according to the instructions provided.

### Anchorage-independent cell growth

The anchorage-independent cell growth assay was performed as described previously^24^. Briefly, the Eagle’s basal medium containing 0.6% agar, 10% FBS, and different concentration of deguelin or gefitinib was loading to a six-well plate as an agar base. NSCLC cells were then suspended and counted at the concentration of 8000 cells/mL with the Eagle’s basal medium containing 10% FBS, 0.3% agar, and various doses of deguelin or gefitinib, followed by overlaid into the six-well plate containing a 0.6% agar base. The cultures were maintained in a 37 °C, 5% CO_2_ incubator for 2 weeks. Colonies were counted using a microscope.

### Lentiviral package and stable lines generation

The EGFR cDNA clones, including WT EGFR, L858R EGFR, L858R/T790M EGFR, and Del E746-A750 EGFR were subcloned into the lentivirus vector (PS100064, Origene) by SgfI and MluI. Lentiviral Packaging Kit (TR30037, Origene) was used for virus package in 293T cells. The Ba/F3 cells were infected with lentivirus together with 8 μg/mL polybrene for 24 h. 48 h later, 1 μg/mL puromycin was added to the cell culture medium and maintained for another 7 days for the selection of stable cell lines.

### In vitro EGFR kinase assay

The recombinant active WT EGFR, L858R EGFR, L858R/T790M EGFR, and Del E746-A750 EGFR were purchased from Millipore. The in vitro EGFR kinase assay was performed as described previously^25^. Briefly, active EGFR (100 ng) was mixed with various doses of deguelin or 100 nM gefitinib. The reaction was incubated with 500 μM angiotensin II for 5 min at room temperature, followed by incubation with 10 μL of ATP mixture (25 mM MgAc and 0.25 μM ATP containing 10 μCi [γ-32P] ATP) for 15 min at 30 °C and then 25 μL of reaction mixture was transferred onto P81 papers. The papers were washed with 0.75% phosphoric acid twice and then with acetone once. The radioactive incorporation was determined using a scintillation counter.

### In vitro pulldown and ATP competition assays

The in vitro pulldown and ATP competition assays were performed as described previously^26^. Deguelin-Sepharose 4B beads were prepared following the protocol provided by GE Healthcare Biosciences. NSCLC cell lysate (500 μg) or an active kinase with different concentrations of ATP was incubated with deguelin-Sepharose 4B beads or Sepharose 4B beads only in reaction buffer (50 mM Tris–HCl (pH 7.5), 150 mM NaCl, 5 mM EDTA, 1 mM DTT, 0.01% Nonidet P-40, 0.02 mM phenylmethylsulfonyl fluoride, 1×protease inhibitor mixture, and 2 μg/mL bovine serum albumin) at 4 °C with gentle rocking overnight, followed by washing with washing buffer (50 mM Tris–HCl (pH 7.5), 150 mM NaCl, 5 mM EDTA, 1 mM DTT, and 0.01% Nonidet P-40, and 0.02 mM phenylmethylsulfonyl fluoride) five times. Protein binding was analyzed by IB.

### Molecular modeling

*Homology modeling*: The three-dimensional structure of EGFR with exon 19 deletion mutation was modeled based on the wild type (WT) crystal structure of EGFR using Modeler^27^. Through extensive analysis of the deposited structures in Protein Data Bank (PDB)^28^, the crystal structure of EGFR (PDB: 4JR3) was used as the template for homology modeling. Ten models were generated and evaluated with the Discrete Optimized Protein Energy (DOPE) score implemented in Modeler. Finally, the best model was adapted for the subsequent docking studies. Molecular docking: After carefully prepared the structures of WT EGFR (PDB: 4JR3), L858R EGFR (PDB: 2ITV), L858R/T790M EGFR (PDB: 3W2P) as well as EGFR with exon 19 deletion, including filling in missing side chains, adding hydrogens and minimizing heavy atoms with default parameters using Protein Preparation Wizard in Schrödinger Suite 2013, the corresponding protein grid files were generated suitable for docking. Then the structure file of the ligand, deguelin, was well pretreated in LigPrep, and docking was performed based on the standard precision mode of Glide with default settings. Docking poses for each receptor–ligand complex were then analyzed for binding modes, and final figures were generated using PyMOL.

### Ubiquitination assay

For endogenous ubiquitination analysis, cells were lysed with modified RIPA buffer containing 1% SDS (20 mM NAP, pH 7.4, 150 mM NaCl, 1% Triton, and 0.5% sodium-deoxycholate), protease inhibitors, and 10 mM N-ethylmaleimide (NEM). The lysates were sonicated for 30 s and boiled at 95 °C for 15 min, then diluted with 0.1% SDS containing RIPA buffer and centrifuged at 16,000×*g* for 15 min. The supernatant was transferred to a new tube and incubated with Mcl-1 antibody plus protein A-Sepharose beads overnight at 4 °C. Beads were washed and subjected to IB analysis. For in vivo ubiquitination assay, cells were lysed with lysis buffer (6 M guanidine–HCl, 0.1 M Na_2_HPO_4_/NaH_2_PO_4_, 0.01 M Tris/HCl, pH 8.0, 5 mM imidazole, and 10 mM β-mercaptoethanol) supplemented with protease inhibitors and 10 mM NEM. After sonication and centrifugation, the supernatant was incubated with 40 μL Ni-NTA-agarose beads (#30210, QIAGEN Inc) at room temperature for 4 h. The beads were centrifuged and washed with the following buffers: (A) 6 M guanidine–HCl, 0.1 M Na_2_HPO_4_/NaH_2_PO_4_, 0.01 M Tris/HCl, pH 8.0, 5 mM imidazole plus 10 mM β-mercaptoethanol; (B) 8 M Urea, 0.1 M Na_2_HPO_4_/NaH_2_PO_4_, 0.01 M Tris/HCl, pH 8.0, 10 mM imidazole, 10 mM β-mercaptoethanol plus 0.1% Triton X-100; (C) 8 M urea, 0.1 M Na_2_HPO_4_/NaH_2_PO_4_, 0.01 M Tris/HCl, pH 6.3, 10 mM β-mercaptoethanol (buffer A), 20 mM imidazole plus 0.2% Triton X-100; (D) 8 M urea, 0.1 M Na_2_HPO_4_/NaH_2_PO_4_, 0.01 M Tris/HCl, pH 6.3, 10 mM β-mercaptoethanol, 10 mM imidazole plus 0.1% Triton X-100; (E) 8 M urea, 0.1 M Na_2_HPO_4_/NaH_2_PO_4_, 0.01 M Tris/HCl, pH 6.3, 10 mM β-mercaptoethanol, 10 mM imidazole plus 0.05% Triton X-100. After the last wash, the beads were boiled with 2×SDS sample loading buffer containing 200 mM imidazole, and the supernatant was separated on an SDS–PAGE, followed by Western blotting.

### In vivo tumor growth

All mice were maintained and manipulated according to strict guidelines established by the Medical Research Animal Ethics Committee, Central South University, China. NSCLC cells, including HCC827 cells (2 × 10^6^), H1975 (1 × 10^6^), A549 (2 × 10^6^) and H3255 (2 × 10^6^) were suspended in 100 μL RPMI-1640 medium and inoculated s.c. into the right flank of 6-week-old female athymic nude mice. Deguelin (3 mg/kg) or vehicle was administrated daily by i.p. injection when the tumor volume reached 100 mm^3^, whereas gefitinib (2 mg/kg) was initiated and repeated daily by oral gavage in dimethyl sulfoxide (5%) and polyethylene glycol (PEG400; 5%) PBS^26^. Mouse body weight was recorded, and tumor volume was determined by caliper. Tumor volume was calculated following the formula of *A* × *B*^2^ × 0.5, wherein *A* is the longest diameter of the tumor, *B* is the shortest diameter, and *B*^2^ is *B* squared.

### Immunohistochemical (IHC) staining

IHC staining was performed as described previously^29^. Briefly, tissue sections from xenograft tumor tissues were baked at 60 °C for 2 h, deparaffinized, and rehydrated. The slide was unmasked by submersion into boiling sodium citrate buffer (10 mM, pH 6.0) for 10 min, and then treated with 3% H_2_O_2_ for 10 min. The slide was blocked with 50% goat serum albumin in 1 × PBS in a humidified chamber for 1 h at room temperature. Primary antibody was incubated at 4 °C in a humidified chamber overnight. After hybridized with the second antibody for 45 min at room temperature, the DAB substrate was used for target protein visualization. Hematoxylin was used for counterstaining. Slides were viewed under a light microscope and analyzed using Image-Pro Plus software (version 6.2) program (Media Cybernetics).

### Statistical analysis

Statistical analyses were performed using SPSS (version 16.0 for Windows, SPSS Inc., Chicago, IL, USA) and GraphPad Prism 5 (GraphPad 5.0, San Diego, CA, USA). The quantitative data were expressed as means ± SD as indicated. Significant differences were determined by the Student *t*-test or ANOVA. A probability value of <0.05 was used as the criterion for statistical significance.

## Results

### Deguelin inhibits the growth of both gefitinib sensitive and resistant NSCLC Cells

**Fig. 1:**
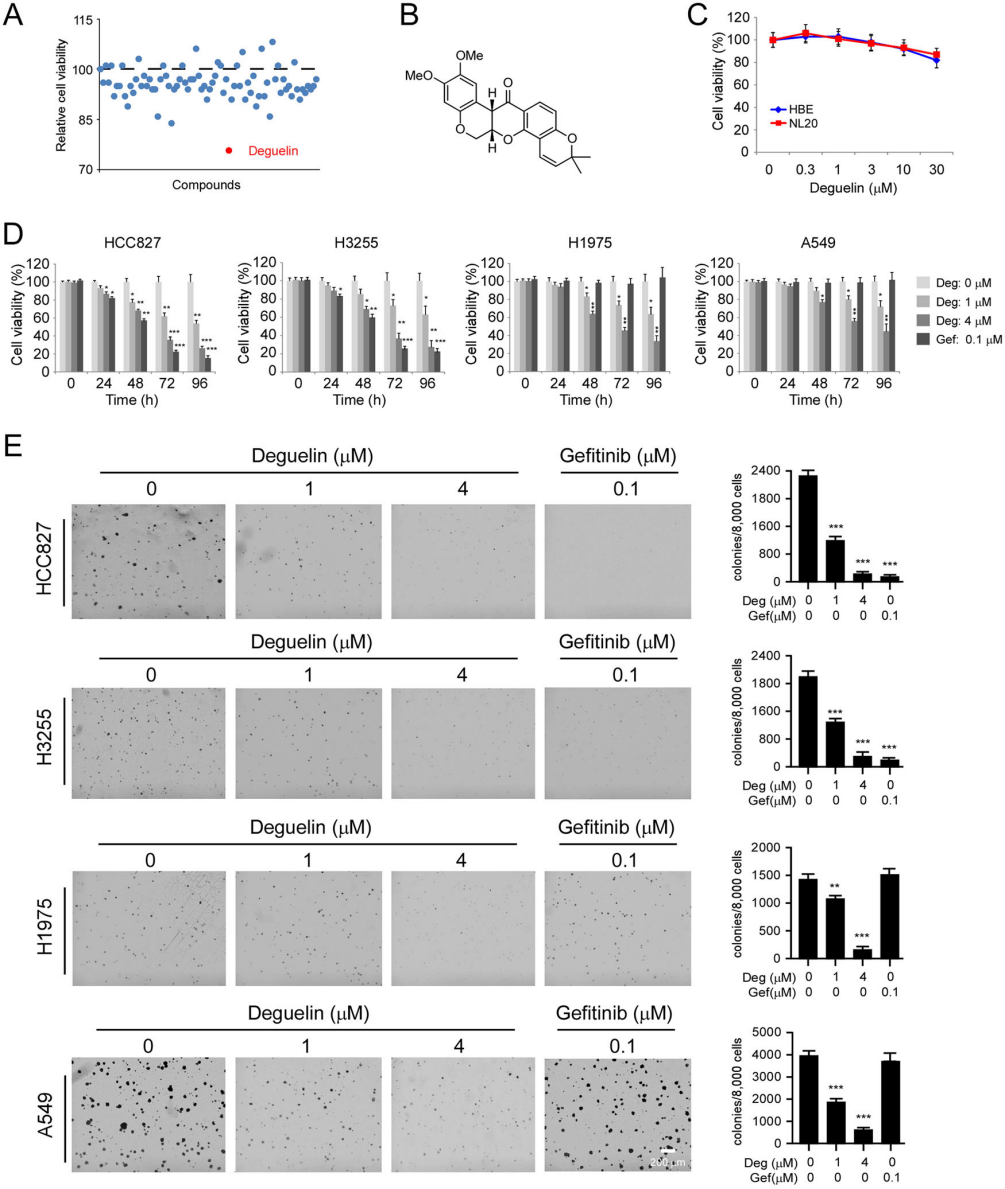
Deguelin is a candidate compound that inhibits non-small cell lung cancer (NSCLC) cells. **a** The inhibitory efficacy of screened compounds on cell viability of HCC827 cells. **b** The structure of deguelin. **c** MTS assay analysis of cell viability in HBE and NL20 cells with deguelin treatment. **d** MTS assay analysis of cell viability in HCC827, H3255, H1975, and A549 cells with deguelin or gefitinib treatment. *p < 0.05, **p < 0.01, ***p < 0.001. **e** Anchorage-independent cell growth of HCC827, H3255, H1975, and A549 cells with deguelin or gefitinib treatment. *p < 0.05, **p < 0.01, ***p < 0.001.

To discover natural compounds (Supplementary Table 1) that can suppress NSCLC cells, we screened a library of 79 natural products using MTS assay. The results showed that only deguelin decreased cell viability over 25% at the concentration of 1 μM (Fig. 1a, b). Importantly, deguelin did not exhibit significant toxicity against normal immortalized lung epithelial cells HBE and NL20 (Fig. 1c). We then determined whether deguelin had any inhibitory effect on anchorage-dependent growth of both gefitinib sensitive and resistant NSCLC Cells. Two gefitinib sensitive cells, HCC827 (EGFR Del E746-A750) and H3255 (EGFR L858R), and two gefitinib-resistant cells H1975 (EGFR L858R/T790M) and A549 (EGFR WT) were used for further study. As shown in Fig. 1d, gefitinib significantly inhibited anchorage-dependent cell growth of HCC827 and H3255 cells time-dependently, whereas no effect of gefitinib was observed on any of these two resistant cell lines H1975 and A549. In contrast, deguelin exhibited a strong inhibitory effect on both gefitinib sensitive and resistant cells in a dose-dependent and time-dependent manner (Fig. 1d). We next determined the anti-tumor effect of deguelin on anchorage-independent cell growth of NSCLC cells. Our data indicated that gefitinib substantially suppressed the colony formation of HCC827 and H3255 as expected, and deguelin could also significantly decrease HCC827 and H3255 cells growth in soft agar at various concentrations (Fig. 1e). Furthermore, results showed that deguelin markedly attenuated colony formation of gefitinib-resistant H1975 and A549 cells dose-dependently, whereas gefitinib failed to do so (Fig. 1e). These results indicate that deguelin suppresses the growth of both gefitinib sensitive and resistant NSCLC cells.

### Deguelin binds and inhibits both WT and mutant EGFR ex vivo and in vitro

**Fig. 2:**
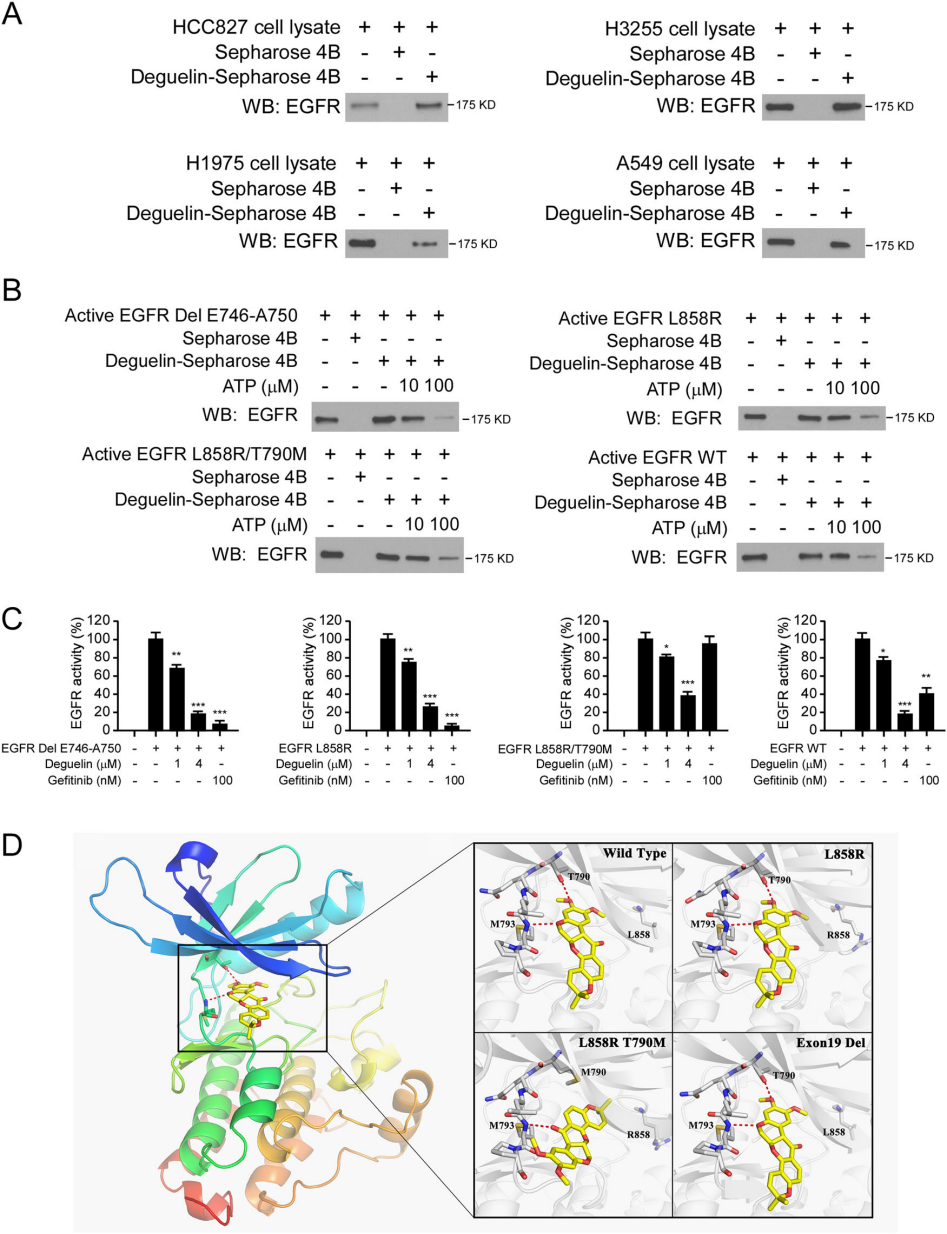
Deguelin inhibits EGFR kinase activity in vitro and ex vivo. **a** Cell lysates (500 μg) from HCC827, H3255, H1975, and A549 cells were incubated with deguelin-Sepharose 4B beads (Sepharose 4B beads only as control) overnight at 4 °C. The beads were washed and boiled with loading buffer and subjected to immunoblotting (IB) analysis. **b** Active EGFR kinases, including EGFR Del E746-A750, EGFR L858R, EGFR L858R/T790M, and EGFR WT, were incubated with different concentrations of ATP overnight, followed by incubation with deguelin-Sepharose 4B beads for 4 h. The beads were washed and boiled with loading buffer, EGFR protein level was detected by IB analysis. **c** Deguelin inhibits WT and mutant EGFR kinase activities in a dose-dependent manner. Gefitinib was used as a positive control. *p < 0.05, **p < 0.01, ***p < 0.001. **d** Binding modes of deguelin with wildtype and mutated EGFRs predicted by molecular docking. Left, a cartoon representation of the deguelin-binding pocket in EGFR. Right, different binding modes of deguelin with four types of EGFR. The ligands were shown in yellow sticks, while proteins were depicted in cartoon representation with key residues indicated as gray sticks and labeled. Hydrogen bonds were shown as red dashed lines.

To better understand the underlying mechanisms of deguelin, we determined whether deguelin could affect EGFR-signaling pathway. First, we detected the binding activity between deguelin and EGFR WT and mutants (Del E746-A750, L858R, L858R/T790M) ex vivo. By incubation with the whole-cell lysates from HCC827, H1975, H3255, and A549 (Fig. 2a), we observed that EGFRs were pulled down by deguelin-conjugated Sepharose 4B beads but not by Sepharose 4B beads alone. This result suggested that deguelin interacts with both WT EGFR and mutant EGFRs. To further validate whether the interaction between deguelin and EGFRs could inhibit the kinase activity of EGFRs, we conducted ATP competition assay with deguelin-conjugated Sepharose 4B beads. The result showed that the binding between deguelin and EGFRs was decreased in the presence of ATP (Fig. 2b), which implied that binding with deguelin might disturb the interaction between EGFRs and ATP. The in vitro EGFR kinase assay showed that both deguelin and gefitinib significantly suppressed EGFR kinase activity in activating mutants, Del E746-A750, and L858R. Deguelin, but not gefitinib, attenuated the kinase activity of EGFR L858R/T790M and WT (Fig. 2c). Moreover, the kinase activity was decreased to <20% in the Del E746-A750 mutant, indicating this mutant is the most sensitive one to deguelin treatment in vitro (Fig. 2c).

Next, we performed the docking model of deguelin toward WT EGFR, L858R single mutant, L858R/T790M mutant, and exon 19 deletion mutant. As shown in Fig. 2d, the docking poses suggested that deguelin could penetrate deeply into the pocket and form a hydrogen bond with the backbone nitrogen of Met793 in the hinge region in all four types of EGFR. Besides, the hydrogen bonding between Thr790 and the methoxyl of deguelin was also crucial. Thus, in the L858R/T790M-mutated EGFR pocket, deguelin might take a different binding mode to avoid a stereo clash with Met790, but it still can form a hydrogen bond with Met793, which might retain the binding affinity. There was little influence on deguelin binding for L858R mutation and exon 19 deletion because Leu858 was far from the ligand, and exon 19 deletion caused marginal variation of the pocket (Fig. 2d). These results suggested that deguelin was a good hit for inhibition of WT and several mutated EGFRs. Overall, our data suggest that deguelin interacts with WT and several mutated EGFRs and inhibits their kinase activity.

**Fig. 3:**
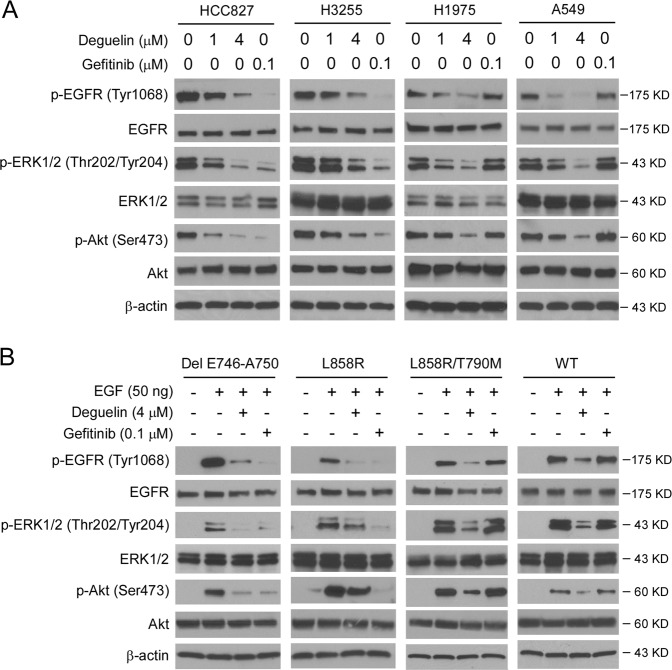
Deguelin suppresses EGFR signaling. **a** Human NSCLC cells, including HCC827, H3255, H1975, and A549 cells, were treated with deguelin and gefitinib, whole-cell extracts (WCE) were collected and subjected to IB analysis using the primary antibodies as indicated. **b** Ba/F3 cells expressing EGFR WT and various EGFR mutants were pre-incubated with deguelin or gefitinib for 2 h followed by treatment with EGF or 15 min in 0.1% FBS containing cell culture medium. WCE was prepared and subjected to IB analysis.

We next examined the effect of deguelin on EGFR signaling in human NSCLC cells, including HCC827, H3255, H1975, and A549. IB analysis indicated that the phosphorylation of EGFR was decreased in response to deguelin treatment in all of these four cell lines (Fig. 3a). Also, deguelin substantially inhibited the phosphorylation of Akt (Ser473) and ERK1/2 (Thr202/Tyr204), well-known downstream targets of EGFR. In contrast, gefitinib only attenuated the activation of EGFR signaling in HCC827 and H3255, but not A549 and H1975 cells (Fig. 3a). To further confirm the effect of deguelin on EGFR signaling, we generated stable cell lines of Ba/F3 cells carrying various EGFRs, including WT, L858R, L858R/T790M, and Del E746-A750 mutants. A similar inhibitory effect of deguelin on EGFR signaling was observed in these stable cell lines (Fig. 3b). However, gefitinib failed to reduce the phosphorylation of EGFR, Akt, and EKR1/2 in L858R/T790M and WT EGFR expression stable cells (Fig. 3b). These data indicate that deguelin inhibits the activation of EGFR signaling in both WT and mutant EGFRs expressing NSCLC cells.

### Deguelin down-regulates Mcl-1 and induces apoptosis in NSCLC cells

**Fig. 4:**
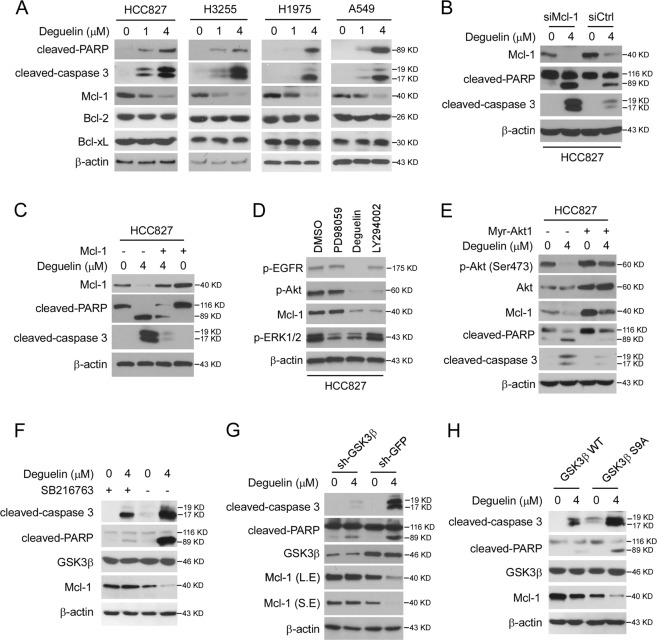
Deguelin attenuates Mcl-1 expression in NSCLC cells. **a** Human NSCLC cells, including HCC827, H3255, H1975, and A549 cells, were treated with deguelin for 24 h, WCE were collected and subjected to IB analysis. **b** HCC827 cells were transfected with siCtrl or siMcl-1, followed by treatment with deguelin for 24 h, WCE were collected and subjected to IB analysis. **c** HCC827 cells were transfected with Mcl-1 construct, followed by treatment with deguelin for 24 h, WCE were collected and subjected to IB analysis. **d** HCC827 cells were treated with DMSO, PD98059, deguelin, or LY294002 for 24 h, WCE were collected and subjected to IB analysis. **e** HCC827 cells were transfected with Myr-Akt1 construct, followed by treatment with deguelin for 24 h, WCE were collected and subjected to IB analysis. **f** HCC827 cells were treated with deguelin or SB216763 for 24 h, WCE were collected and subjected to IB analysis as indicated. **g** HCC827 cells stable expression sh-GFP or sh-GSK3β were treated with deguelin or DMSO for 24 h, WCE were collected and subjected to IB analysis as indicated. **h** HCC827 cells were transfected with GSK3β WT or GSK3β S9A construct, followed by treatment with deguelin for 24 h, WCE was collected and subjected to IB analysis.

EGFR signaling plays a crucial role in maintaining cell growth and survival in NSCLC cells. Based on our previous data, we next examined whether deguelin affects cell apoptosis. Deguelin promoted the expression of cleaved-PARP and -caspase 3 in all of these treated cells (Fig. 4a), indicating the activation of apoptosis signaling. Deguelin substantially reduced the protein level of Mcl-1, but not the Bcl-2 and Bcl-xL. Furthermore, knockdown of Mcl-1 markedly enhanced the protein level of deguelin-induced cleaved-PARP and -caspase 3 (Fig. 4b), whereas ectopic overexpression of Mcl-1 compromised this process (Fig. 4c), suggesting that decrease of Mcl-1 is required for restoring deguelin sensitivity. To further determine which downstream signaling regulates Mcl-1 expression, HCC827 cells were treated with two kinase inhibitors, PD98059 and LY294002. IB analysis showed that inhibition of Akt, but not ERK1/2, resulted in a robust decrease of Mcl-1 (Fig. 4d). Moreover, overexpression of constitutively activated Akt1 (Myr-Akt) impaired deguelin-induced Mcl-1 reduction, and cleaved-PARP and -caspase 3 induction (Fig. 4e). Early studies showed that GSK3β-mediated Mcl-1 phosphorylation promoted Mcl-1 ubiquitination and destruction by 26S proteasome^30^. Our data showed that deguelin promoted Mcl-1 phosphorylation at S159, which accompanied by decreasing of Mcl-1 protein level in all of these four NSCLC cells (Supplementary Fig. 1). To determine whether deguelin decreased Mcl-1 is dependent on GSK3β, we blocked the kinase activity of GSK3β by small molecule inhibitor SB216763. The result showed that suppression of GSK3β attenuated deguelin-induced Mcl-1 reduction (Fig. 4f). Likewise, the silencing of GSK3β by shRNA restored Mcl-1 protein level and compromised deguelin-induced apoptosis (Fig. 4g). GSK3β is a downstream kinase of the Akt signaling whose activity is inhibited by Akt-mediated phosphorylation at Ser9. Using the Ser9 to Ala9 (S9A) mutant, we further found that activation of GSK3β promoted deguelin-induced Mcl-1 reduction and enhanced the expression of cleaved-PARP and -caspase 3 (Fig. 4h). Our data support the notion that deguelin down-regulates Mcl-1 in an Akt-GSK3β signaling-dependent manner.

### Deguelin promotes Mcl-1 ubiquitination and degradation

**Fig. 5:**
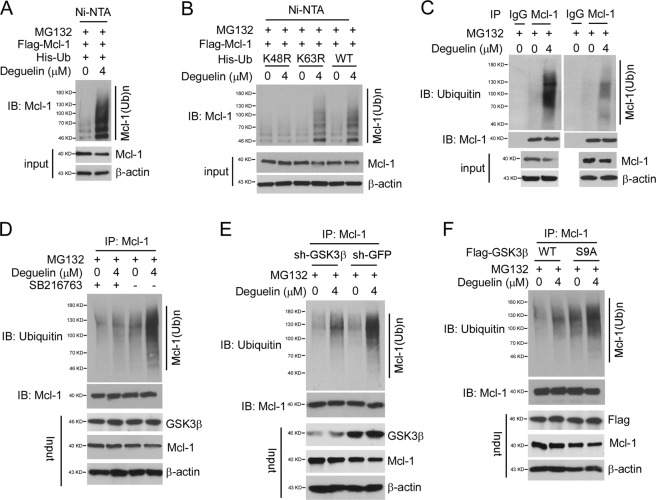
Deguelin promotes Mcl-1 ubiquitination in NSCLC cells. **a** HCC827 cells were transfected with various constructs as indicated and treated with deguelin for 24 h, then incubated with MG132 for another 6 h. WCE were harvested and subjected to in vivo ubiquitination assay. **b** HCC827 cells were transfected with various His-Ub mutant constructs as indicated. MG132 was added to the cell culture medium for 6 h before harvest. WCE was subjected to in vivo ubiquitination assay. **c** HCC827 (left) and H1975 (right) cells were treated with DMSO or deguelin for 24 h, followed by incubation with MG132 for another 6 h. WCE was collected and subjected to endogenous Mcl-1 ubiquitination analysis. **d** HCC827 cells were treated with deguelin or/and SB216763 for 24 h, followed by incubation with MG132 for another 6 h. WCE was collected and subjected to endogenous Mcl-1 ubiquitination analysis. **e** HCC827 sh-GFP and sh-GSK3β stable cells were treated with DMSO or deguelin for 24 h, followed by incubation with MG132 for another 6 h. WCE was harvested and subjected to endogenous ubiquitination analysis. **f** HCC827 cells were transfected with GSK3β WT or GSK3β S9A construct, treated with DMSO or deguelin for 24 h, followed by incubation with MG132 for another 6 h. WCE was harvested and subjected to endogenous ubiquitination analysis.

Because activation of GSK3β is required for deguelin-induced Mcl-1 downregulation, we hypothesized that deguelin might promote Mcl-1 ubiquitination and degradation. The in vivo ubiquitination assay showed that treatment with deguelin increased Mcl-1 polyubiquitination robustly (Fig. 5a). Using His-Ub K48R (Lysine 48 to Arginine) and K63R mutants, we demonstrated that deguelin-promoted Mcl-1 ubiquitination was K48-, but not K63-linked polyubiquitination chains (Fig. 5b). The endogenous Mcl-1 ubiquitination was also found to be enhanced with deguelin treatment in HCC827 and H1975 cells (Fig. 5c). Because inhibition of GSK3β by small molecular inhibitor SB216763 attenuated the deguelin-induced reduction of Mcl-1 protein (Fig. 4f), we further examined whether SB216763 regulates Mcl-1 ubiquitination. The result showed that treatment with SB216763 impaired deguelin-induced Mcl-1 ubiquitination as expected (Fig. 5d). Likewise, deguelin-mediated Mcl-1 ubiquitination was decreased in GSK3β knockdown cells (Fig. 5e). Mutation of GSK3β deactivation site, Ser9, enhanced Mcl-1 ubiquitination with deguelin treatment (Fig. 5f). In contrast, suppression of GSK3β kinase activity by Myr-Akt1 reversed this process (Supplementary Fig. 2a) and attenuated deguelin-mediated decrease of cell viability and colony formation (Supplementary Fig. 2b, c). These results suggest that GSK3β plays a critical role in deguelin-promoted Mcl-1 ubiquitination and destruction.

### FBW7 is required for deguelin-induced Mcl-1 ubiquitination

**Fig. 6:**
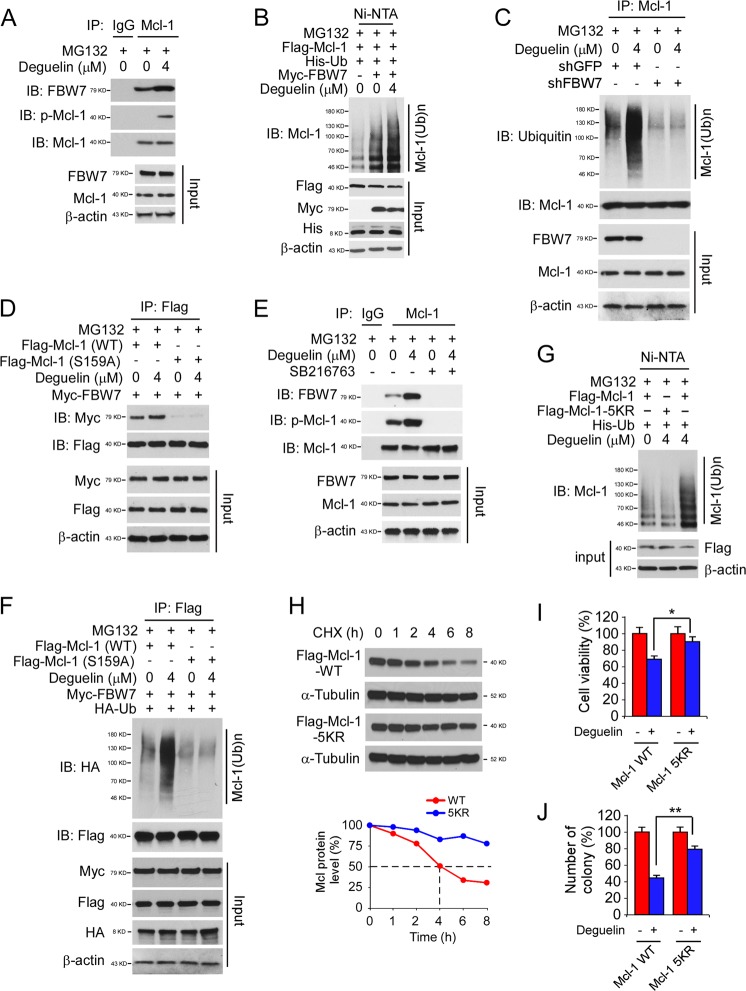
FBW7 is required for deguelin-induced Mcl-1 down-regulation. **a** HCC827 cells were treated with deguelin or DMSO for 24 h, followed by incubation with MG132 for another 6 h. Cell lysates were subjected to co-immunoprecipitation (co-IP) analysis. **b** HCC827 cells were transfected with various constructs and treated with deguelin or DMSO for 24 h, followed by incubation with MG132 for another 6 h. WCE was subjected to in vivo ubiquitination assay. **c** HCC827 sh-GFP and sh-FBW7 stable cells were treated with DMSO or deguelin for 24 h, followed by incubation with MG132 for another 6 h. WCE was harvested and subjected to endogenous ubiquitination analysis. **d** HCC827 cells were transfected with various constructs and treated with deguelin for 24 h, followed by incubation with MG132 for another 6 h. Cell lysates were subjected to co-IP analysis. **e** HCC827 cells were treated with deguelin or/and SB216763 for 24 h, followed by incubation with MG132 for another 6 h. Cell lysates were subjected to co-IP analysis. **f** HCC827 cells were transfected with various constructs and treated with deguelin for 24 h, followed by incubation with MG132 for another 6 h. Cell lysates were subjected to endogenous ubiquitination analysis. **g** HCC827 cells were transfected with various constructs and treated with deguelin for 24 h, followed by incubation with MG132 for another 6 h. Cell lysates were subjected to in vivo ubiquitination analysis. **h** HCC827 cells were transfected with various constructs and treated with cycloheximide (CHX). WCE was harvested at different time points and subjected to IB analysis. **i, j** Cell viability **i**, and colony formation **j** of HCC827 cells transfected with Mcl-1 WT or Mcl-1 5KR and treated with deguelin. *p < 0.05, **p < 0.01.

Previous studies suggested that the E3 ligase SCF^Fbw7^ promotes Mcl-1 ubiquitination in a phosphorylation-dependent manner^31,32^. Our data showed that the phosphorylation of Mcl-1 at S159 was increased with deguelin treatment (Supplementary Fig. 1). Thus, we speculated that FBW7 might require for Mcl-1 ubiquitination. To validate this hypothesis, we first examined the interaction between Mcl-1 and FBW7. The result showed that FBW7 bound with Mcl-1 in HCC827 cells, and this interaction was elevated in the presence of deguelin (Fig. 6a). Mcl-1 polyubiquitination also increased when FBW7 was simultaneously overexpressed and further enhanced by deguelin treatment (Fig. 6b). Moreover, the endogenous Mcl-1 ubiquitination was reduced markedly in FBW7 shRNA stable expression HCC827 cells (Fig. 6c). Consistently, knockdown of FBW7 impaired deguelin-mediated decrease of cell viability and colony formation in HCC827 and H1975 cells (Supplementary Fig. 3a,b). An early study demonstrated that phosphorylation of S159 is required for Mcl-1 binding with FBW7^31^. Indeed, our data showed that mutation of S159 compromised the interaction between Mcl-1 and FBW7 even with deguelin treatment (Fig. 6d). Moreover, pharmacological inactivation of the protein kinase GSK3β decreased Mcl-1 phosphorylation on S159 and reduced the interaction with FBW7 (Fig. 6e). The in vivo ubiquitination result showed that deguelin promoted the ubiquitination of WT Mcl-1, but not the S159A mutant (Fig. 6f), further confirm that phosphorylation of Mcl-1 by GSK3β is essential for deguelin-induced Mcl-1 downregulation.

Human Mcl-1 protein contains a total of 13 lysine residues, and 5 lysine residues, including K5, K40, K136, K194, and K197, have been shown to be ubiquitinated by FBW7^31^. To determine whether deguelin-induced Mcl-1 ubiquitination occurs on these lysine sites, we constructed a 5KR mutant, in which all of these five lysine residues were mutated to arginine. The in vivo ubiquitination result showed that deguelin-induced Mcl-1 ubiquitination was reduced markedly in the Mcl-1 5KR mutant (Fig. 6g). Consistently, the half-life of Mcl-1 5KR was extended substantially than that of Mcl-1 WT (Fig. 6h). Ectopic overexpression of Mcl-1 5KR conferred resistant to deguelin, the cell viability (Fig. 6i), and colony number (Fig. 6j) were significantly increased when compared with those of Mcl-1 WT. The in vivo xenograft model showed that overexpression of Mcl-1 5KR mutant reduced the anti-tumor efficacy of deguelin (Supplementary Fig. 4a–c). The tumor volume and tumor weight in the deguelin-treated Mcl-1 5KR mutant group were significantly larger than that of the deguelin-treated Mcl-1 WT group (Supplementary Fig. 4a, c). These results indicate that FBW7 is required for deguelin-induced Mcl-1 ubiquitination in NSCLC cells.

### Deguelin suppresses both gefitinib-sensitive and -resistant xenograft tumors growth

**Fig. 7:**
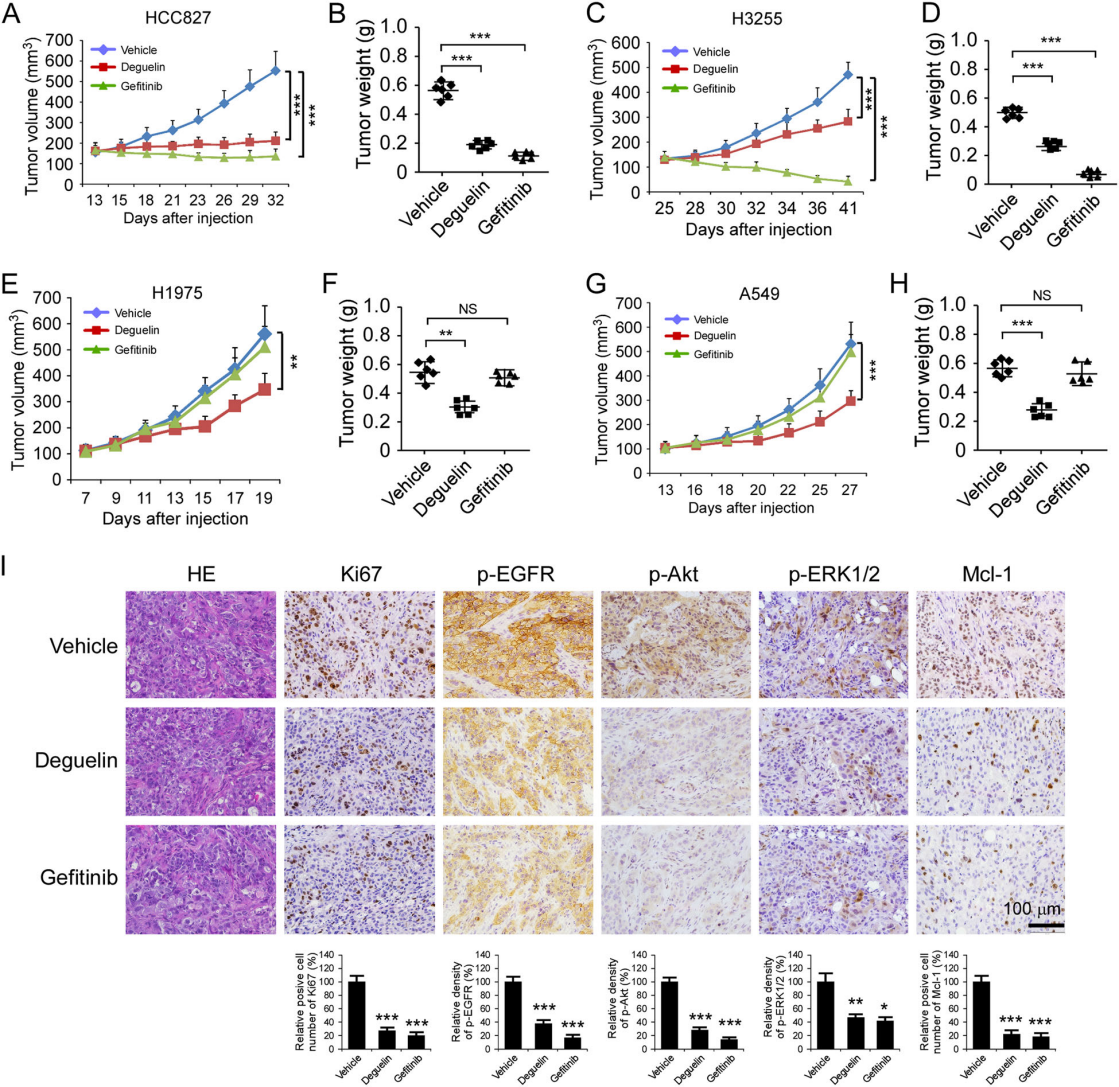
Deguelin inhibits tumor growth in vivo. **a**, **b** Tumor volume **a** and tumor weight **b** of HCC827 xenograft tumors treated with vehicle control, deguelin, or gefitinib. **c**, **d** Tumor volume **c** and tumor weight **d** of H3255 xenograft tumors treated with vehicle control, deguelin, or gefitinib. **e**, **f** Tumor volume **e**, and tumor weight **f** of H1975 xenograft tumors treated with vehicle control, deguelin, or gefitinib. **g**, **h** Tumor volume **g**, and tumor weight **h** of A549 xenograft tumors treated with vehicle control, deguelin, or gefitinib. **i** Immunohistochemistry staining analysis of Ki67, p-EGFR, p-Akt, p-ERK1/2, and Mcl-1 in HCC827 xenograft tumors. *p < 0.05, **p < 0.01, ***p < 0.001.

To explore the anti-tumor activity of deguelin in vivo, we performed mice xenograft models using WT EGFR and mutant EGFR expressing NSCLC cells, including A549, HCC827, H3255, and H1975 cells. When tumor volume reached 100 mm^3^, treatment with deguelin, gefitinib, or vehicle control was initiated. The results showed that once-daily dosing of deguelin significantly inhibited the tumor growth in EGFR-activating mutant-harbored cells, including HCC827 (Fig. 7a, b), H3255 (Fig. 7c, d), and H1975 (Fig. 7e, f) xenograft tumors (Supplementary Fig. 5a–c). Likewise, deguelin delayed the in vivo tumor growth of A549 cells at the same dose (Fig. 7g, h, Supplementary Fig. 5d). However, gefitinib only suppressed tumor growth in the HCC827 and H3255 xenograft models as expected (Fig. 7a–d). In EGFR WT A549 and L858R/T790M mutant H1975 xenograft tumors, gefitinib had no visible effect on tumor growth, whereas deguelin markedly reduced tumor size at the treatment endpoint (Fig. 7e–h). Moreover, IHC analysis was conducted to evaluate the protein levels of Ki67, p-EGFR, p-Akt, p-ERK1/2, and Mcl-1 in HCC827 xenograft tumors. As data shown in Fig. 7i, both deguelin and gefitinib decreased the expression of Ki67 and Mcl-1. Furthermore, deguelin and gefitinib exhibited the inhibitory effect on the activation of EGFR signaling, as the phosphorylation of EGFR, Akt, and ERK1/2 were attenuated when compared to the vehicle-treated xenograft tumors (Fig. 7i). We observed a similar inhibitory effect of deguelin on these proteins in H1975 xenograft tumors (Supplementary Fig. 5e). These results suggest that deguelin exerts a substantial chemotherapeutic effect to overcome gefitinib-resistant xenograft growth in vivo through the inhibition of EGFR signaling and decrease of Mcl-1 expression. To evaluate the in vivo toxicity of deguelin, we monitored mouse body weight with deguelin treatment. Results showed that treatment with deguelin or gefitinib was well-tolerated without significant weight loss (Supplementary Fig. 6a). Blood analysis revealed that HCC827 tumor-bearing mice treated with deguelin did not cause a reduction in RBC and WBC counts (Supplementary Fig. 6b). Consistently, deguelin exhibited no significant toxicity to vital organ functions as measured by the results of kidney, liver, and bone marrow function tests (AST, ALT, BUN, and Hb, Supplementary Fig. 6b).

## Discussion

NSCLC is a leading cause of cancer-related death in the world. Each year, over 1 million new cases of NSCLC are diagnosed, and only <25% patient is suitable for surgical treatment^33^. EGFR-targeted therapy has emerged as the first-line chemotherapeutic method for EGFR-activating mutations harbored advanced NSCLC patients. However, only a few fractions of NSCLC patients respond to TKI treatment, primary and acquired resistance are key clinical barriers to further improving outcomes of targeted therapy^34^. Thus, identification of novel small molecule inhibitors to overcome primary or acquired TKI resistance is still an urgent need in NSCLC treatment. In the present study, we unexpectedly identified a natural product, deguelin, as a potential EGFR inhibitor. Using the in vitro, ex vivo, and in vivo models, we demonstrated that deguelin suppressed the activation of WT and mutant EGFRs, as well as downstream target kinases ERK1/2 and Akt. Deguelin exhibited anti-tumor effects almost equivalent to many approved anticancer agents and showed no significant cytotoxicity with lower in vivo dose.

Mcl-1 is a Bcl-2 family member that suppression the intrinsic apoptotic pathway by modulating mitochondrial integrity^35,36^. Overexpression of Mcl-1 is frequently observed in human cancers, including NSCLC^37^, colorectal^38^, liver^39^, prostate cancer^40^, and multiple myeloma^41^. High level of Mcl-1 often related to poor prognosis and conferred resistance to chemo/radiotherapy^42–47^. A recent study showed that TWEAK stimulation of NSCLC cells induced NF-κB-dependent Mcl-1 protein expression and conferred Mcl-1-dependent chemo- and radioresistance^48^. Furthermore, human T-cell leukemia virus 1 (HTLV-1) Tax protein stabilizes Mcl-1 via TRAF6-dependent K63-linked polyubiquitination to promote cell survival and transformation^49^. Degradation of Mcl-1 by β-TrCP mediates glycogen synthase kinase 3-induced tumor suppression and chemosensitization^30^. Also, Mcl-1 degradation is required for targeted therapeutics to eradicate colon cancer cells^50^. The evidence suggested Mcl-1 might be a promising therapeutic target for cancer treatment. However, so far, there were no Mcl-1 inhibitors have been approved successfully, and the development of alternative approaches to target Mcl-1 is still an urgent demand for cancer treatment. Our data revealed that deguelin decreased the protein level of Mcl-1 in a ubiquitination degradation-dependent manner. Deguelin inhibited the EGFR-Akt signaling in EGFR WT and mutant cell lines, which in turn activated GSK3β and eventually promoted FBW7-mediated Mcl-1 ubiquitination and destruction.

Unlike other Bcl-2 members, Mcl-1 is very unstable, and Mcl-1 abundance is tightly controlled in multiple levels, including transcriptional, posttranscriptional, and posttranslational processes. Recently, ubiquitination and deubiquitination have been demonstrated to play critical roles in controlling Mcl-1 stabilization^51^. Four different E3 ubiquitin-ligases, including FBW7^52^, β-Trcp^30^, Mule^53^, and Trim17^54^, were identified as negative regulators to induce Mcl-1 degradation, whereas the deubiquitinase, such as USP9X^55^, DUB3^56^, and USP13^57^, were required for Mcl-1 deubiquitination and stabilization. In the present study, we demonstrated that deguelin-induced Mcl-1 degradation was E3 ligase FBW7-dependent. Deguelin-inhibited EGFR-Akt signaling, which resulted in activation of GSK3β and eventually promoted GSK3β-mediated Mcl-1 phosphorylation. Furthermore, Mcl-l phosphorylation enhanced the interaction between FBW7 and Mcl-1, which facilitated FBW7-induced Mcl-1 ubiquitination and degradation. Recently, accumulating evidence indicate that destruction of Mcl-1 enhanced TKI sensitivity. Degradation of Mcl-1 by bufalin reverses acquired resistance to osimertinib in EGFR-mutant lung cancer^58^, and modulation of MEK/ERK-dependent Bim and Mcl-1 degradation overcoming acquired resistance to osimertinib^59^. Additionally, depletion of FBW7 results in upregulation of Mcl-1 protein level and confers TKI resistance in PC9 cells^60^, indicating that reduction of Mcl-1 protein level as a strategy to overcome TKI resistance in NSCLC treatment.

Collectively, our study reports that the natural product deguelin inhibits the EGFR–Akt axis, and enhances Mcl-1 ubiquitination and degradation in an FBW7-dependent manner. Targeting FBW7-mediated Mcl-1 destruction is a promising strategy to kill NSCLC cells with either WT or mutant EGFR. Our result could provide a new option for the pre-clinical treatment of NSCLC.

## Supplementary information

Figure S1

Figure S2

Figure S3

Figure S4

Figure S5

Figure S6

Supplementary Table 1

20200206 Revised supplementary figure and table legends
