# Supplementary figures and images for: Deguelin suppresses non-small cell lung cancer by inhibiting EGFR signaling and promoting GSK3β/FBW7-mediated Mcl-1 destabilization
Source: Cell Death Dis. 2020 Feb 21;11(2):143. doi: 10.1038/s41419-020-2344-0 (PMC7035355; doi:10.1038/s41419-020-2344-0)

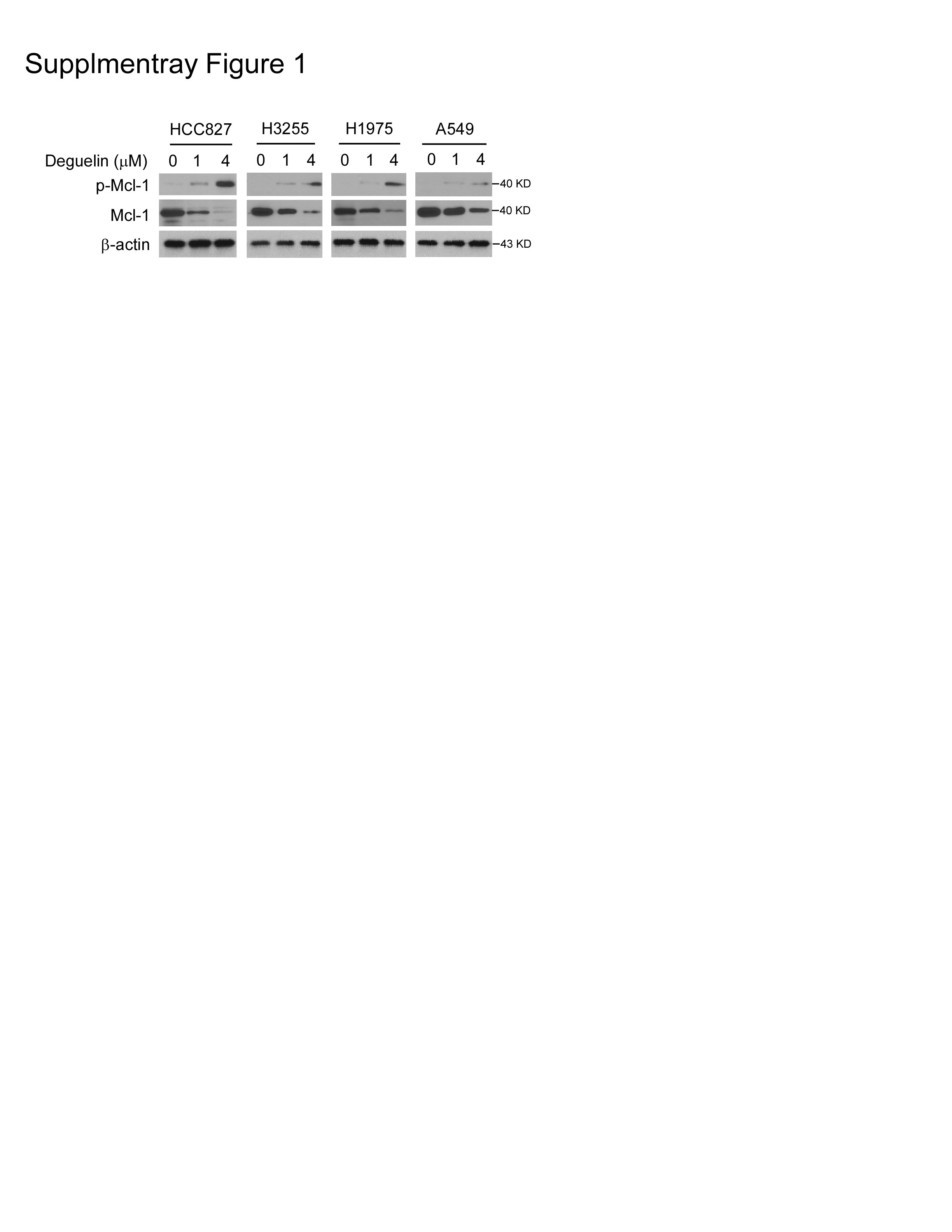

Supplement: Supplementary file 1 — Figure S1 [file 41419_2020_2344_MOESM1_ESM.png]

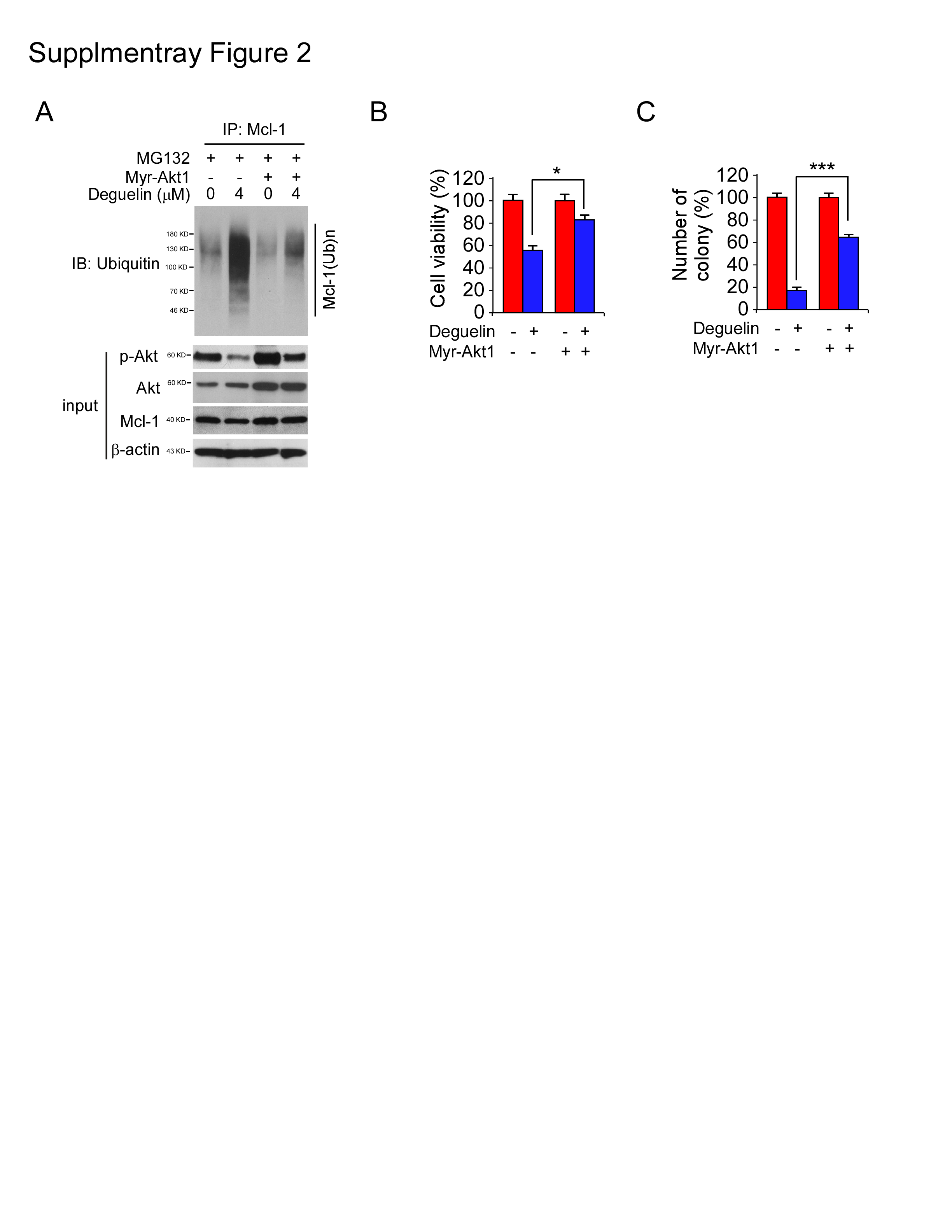

Supplement: Supplementary file 2 — Figure S2 [file 41419_2020_2344_MOESM2_ESM.png]

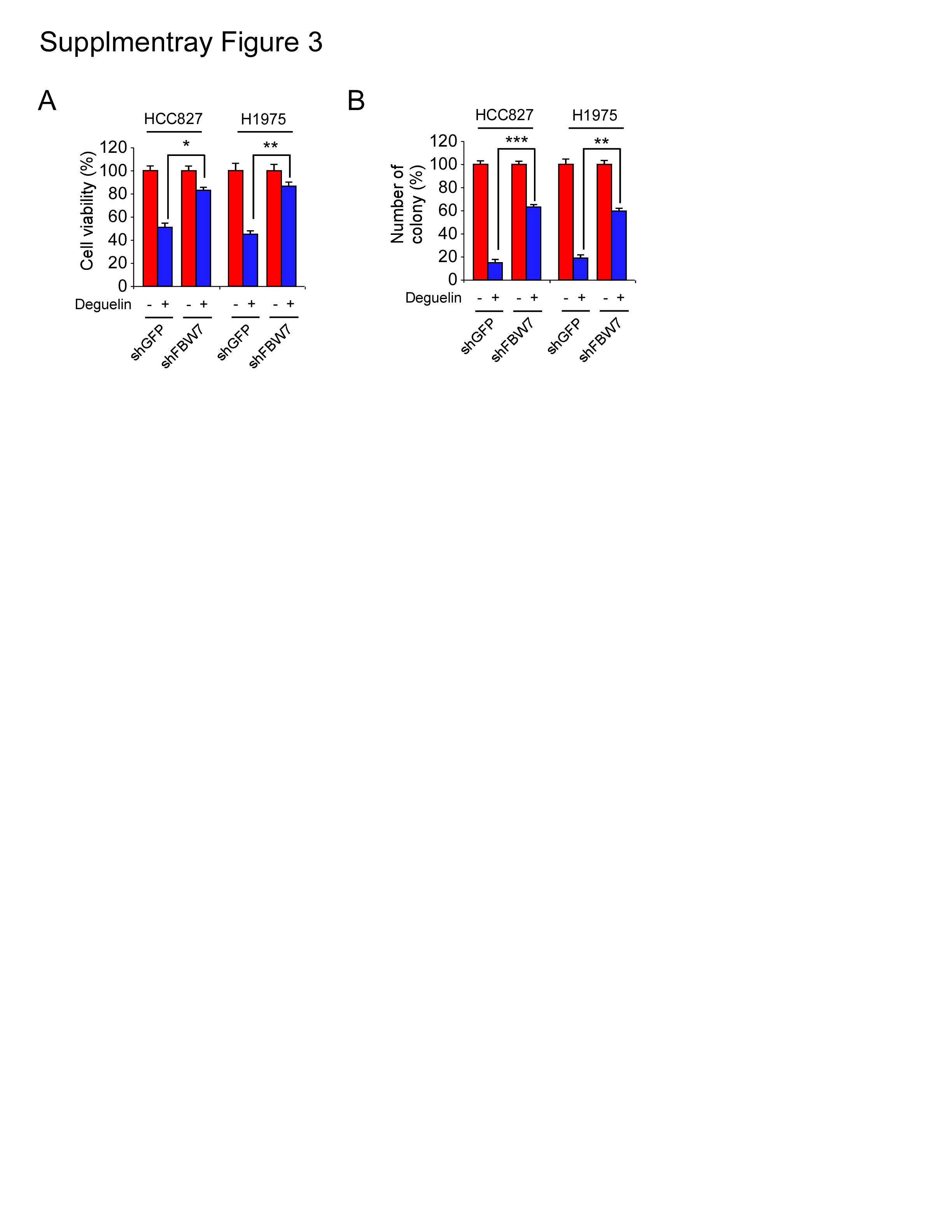

Supplement: Supplementary file 3 — Figure S3 [file 41419_2020_2344_MOESM3_ESM.png]

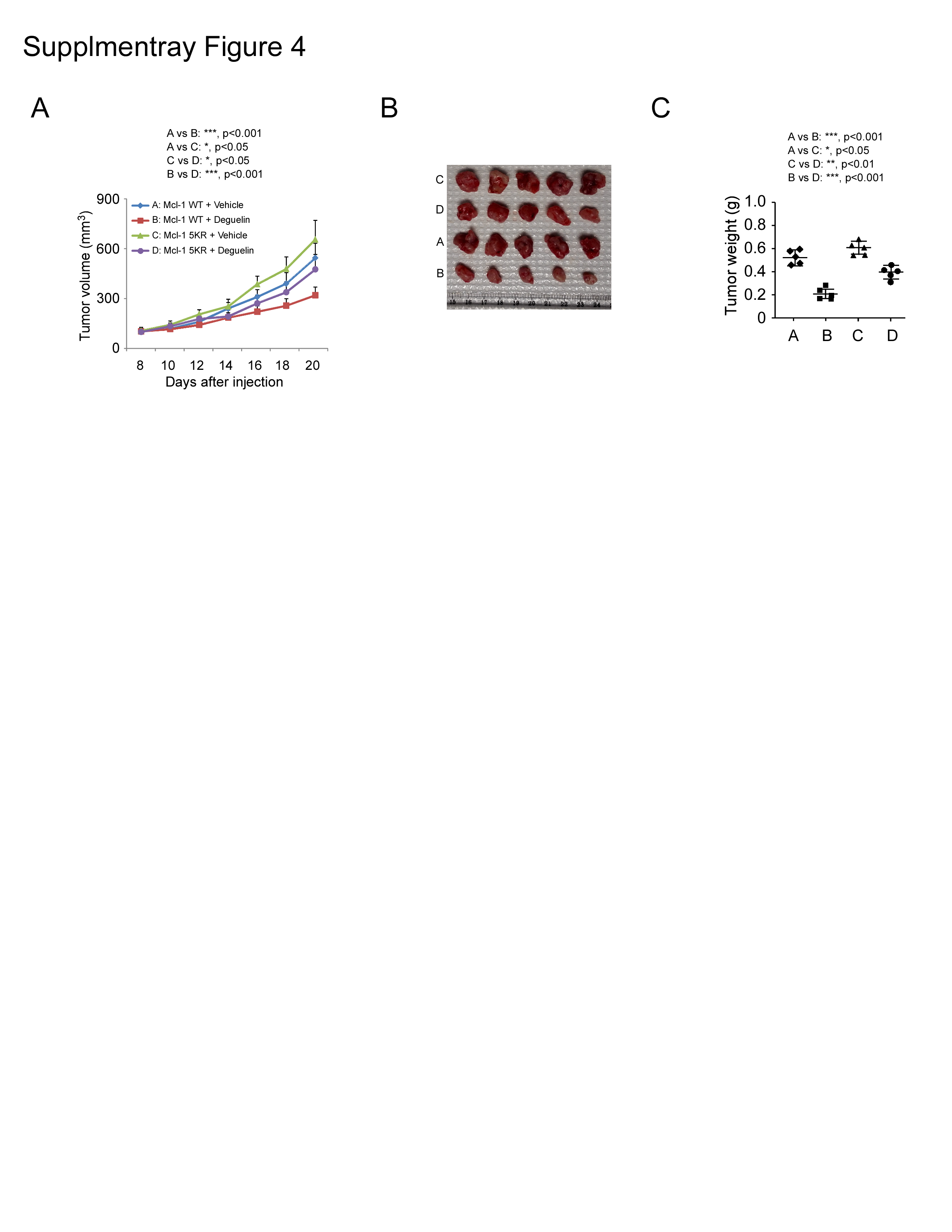

Supplement: Supplementary file 4 — Figure S4 [file 41419_2020_2344_MOESM4_ESM.png]

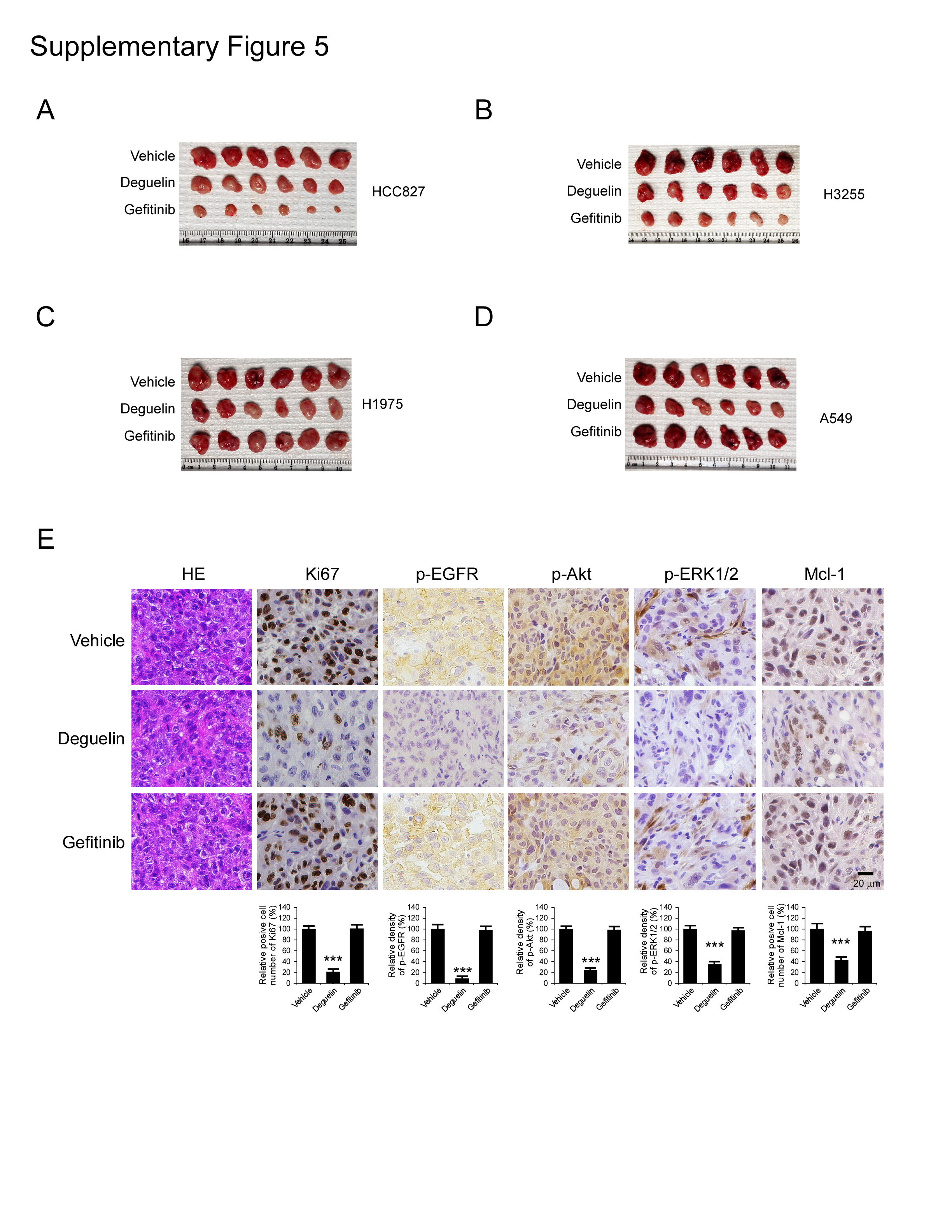

Supplement: Supplementary file 5 — Figure S5 [file 41419_2020_2344_MOESM5_ESM.jpg]

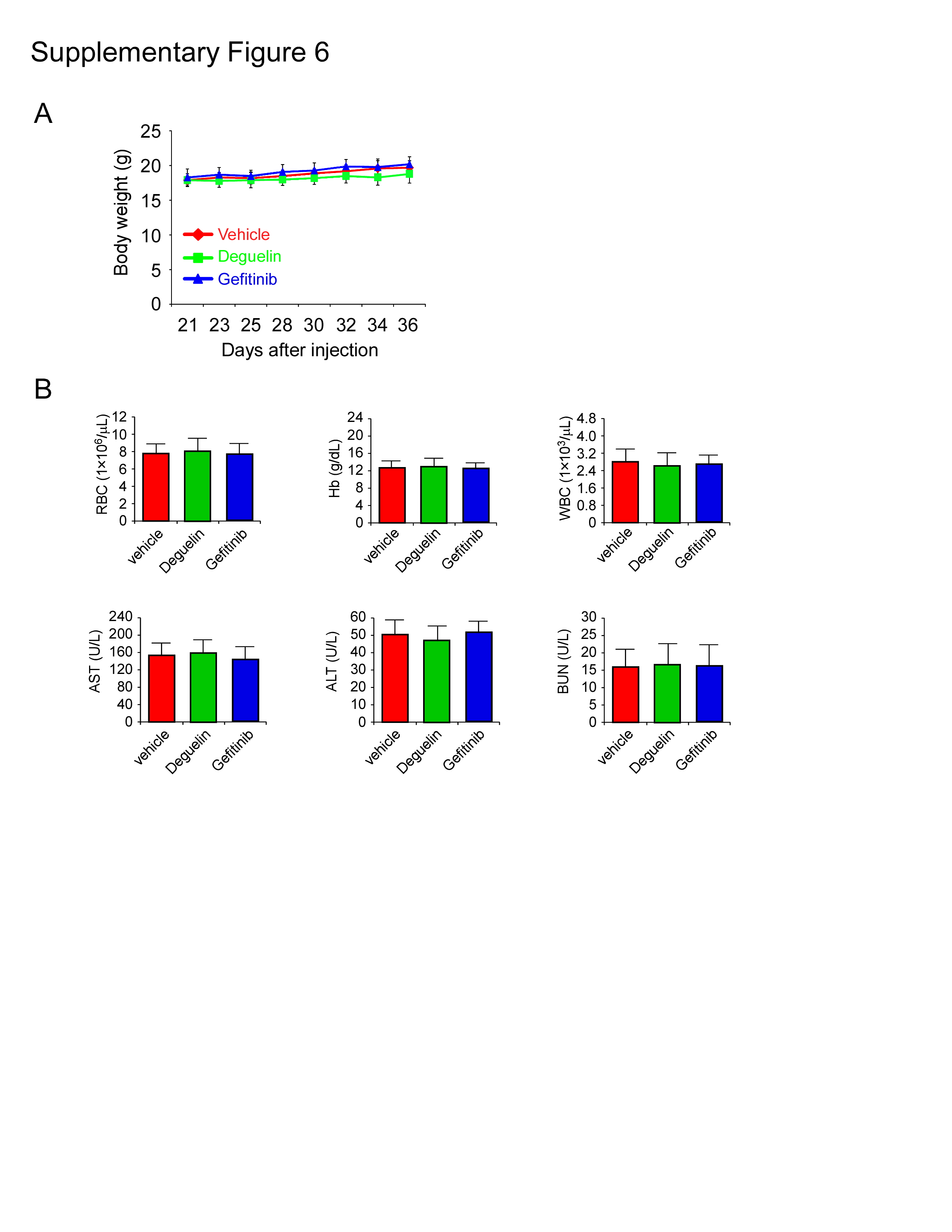

Supplement: Supplementary file 6 — Figure S6 [file 41419_2020_2344_MOESM6_ESM.jpg]
