# Supplementary material for: Deguelin suppresses non-small cell lung cancer by inhibiting EGFR signaling and promoting GSK3β/FBW7-mediated Mcl-1 destabilization
Source: Cell Death Dis. 2020 Feb 21;11(2):143. doi: 10.1038/s41419-020-2344-0 (PMC7035355; doi:10.1038/s41419-020-2344-0)
Supplement: Supplementary file 8 — 20200206 Revised supplementary figure and table legends [file 41419_2020_2344_MOESM8_ESM.docx]

**Supplementary figure legends**

**Fig. 1 Deguelin promotes Mcl-1 phosphorylation at Ser159.** Human NSCLC cells, including HCC827, H3255, H1975, and A549 cells, were treated with deguelin for 24 h, WCE were collected and subjected to IB analysis.

**Fig. 2 Myr-Akt1 attenuates deguelin-induced Mcl-1 ubiquitination. a** HCC827 cells were transfected with Myr-Akt1 and treated with deguelin for 24 h, followed by incubation with MG132 for another 6 h. Cell lysates were subjected to endogenous ubiquitination analysis. **b, c** Cell viability (**b**), and colony formation (**c**) of HCC827 cells transfected with Myr-Akt1 and treated with deguelin. **p*<0.05, ****p*<0.001.

**Fig. 3 Knockdown of FBW7 impairs deguelin-mediated suppression of cell viability and colony formation. a, b** HCC827 and H1975 cells stable expression of sh-GFP or sh-FBW7 were treated with deguelin or DMSO, cell viability (**a**) and colony formation (**b**) were analyzed. **p*<0.05, ***p*<0.01, ****p*<0.001.

**Fig. 4. Mcl-1 5KR mutation attenuates the anti-tumor effect of deguelin *in vivo*. a** Tumor volume of Mcl-1 WT or Mcl-1 5KR overexpressed H1975-derived xenograft tumors treated with vehicle control or deguelin. **b** Photographs of tumors from (**a**). **c** Tumor weight of Mcl-1 WT or Mcl-1 5KR overexpressed H1975-derived xenograft tumors treated with vehicle control or deguelin. **p*<0.05, ***p*<0.01, ****p*<0.001.

**Fig. 5 Deguelin inhibits tumor growth *in vivo*. a-d** Photographs of tumors from HCC827 (**a**), H3255 (**b**), H1975 (**c**), and A549 (**d**) xenograft mouse models with vehicle, deguelin, or gefitinib treatment. **e** Immunohistochemistry staining analysis of Ki67, p-EGFR, p-Akt, p-ERK1/2, and Mcl-1 in H1975 xenograft tumors. ****p*<0.001.

**Fig. 6 Toxicity analysis for treatment with deguelin. a** Body weight of HCC827 xenograft tumor bearing mice with vehicle, deguelin, or gefitinib treatment. **b** Blood analysis of mice with vehicle, deguelin, or gefitinib treatment.

**Supplementary Table legends**

Table 1. Screened compound list.
